# Non-pharmacological options for managing chronic musculoskeletal pain in children with pediatric rheumatic disease: a systematic review

**DOI:** 10.1007/s00296-018-4136-8

**Published:** 2018-08-23

**Authors:** Linde N. Nijhof, Merel M. Nap-van der Vlist, Elise M. van de Putte, Annet van Royen-Kerkhof, Sanne L. Nijhof

**Affiliations:** 1 Department of Pediatrics, Wilhelmina Children’s Hospital, University Medical Centre Utrecht, HP KE.04.133.1, Post box 85090, 3508 AB Utrecht, The Netherlands; 2 Department of Pediatric Rheumatology and Immunology, Wilhelmina Children’s Hospital, University Medical Center Utrecht, Utrecht, The Netherlands

**Keywords:** Rheumatic diseases, Musculoskeletal pain, Chronic pain, Autoimmune diseases, Pediatrics, Juvenile idiopathic arthritis

## Abstract

In patients with a pediatric rheumatic disease (PRD), chronic musculoskeletal pain (CMP) can have a major impact on functioning and social participation. Because CMP is not always alleviated solely by the use of pharmacological approaches, the aim was to systematically review the available evidence regarding non-pharmacological treatment options for reducing CMP in patients with PRD. PubMed, Embase, PsycINFO, and the Cochrane Library were systematically searched for (non-)randomized trials investigating non-pharmacological treatments for CMP in PRD published through October 25, 2017. The GRADE approach was used to assess the quality of evidence. The search yielded 11 studies involving 420 children 5–18 years of age. All studies were relatively small and short-term, and the quality of evidence ranged from very low to moderate. The main modalities within non-pharmacology therapy were psychological interventions and exercise-based interventions. Some studies show modest positive short-term results for psychological and exercise-based interventions. Psychological and exercise-based interventions can have a modest positive result in PRD, with no evidence of side effects. Non-pharmacological therapies are a promising option to alleviate pain in PRD and improve functioning, which can be used as an alternative for or in addition to pharmacological therapies. Because chronic pain can differ etiologically from acute pain in PRD, non-pharmacological therapies might have different effects in patients with or without active inflammation. To best determine the effect of non-pharmacological therapies, future studies should take this difference into account.

**Electronic supplementary material:**

The online version of this article (10.1007/s00296-018-4136-8) contains supplementary material, which is available to authorized users.

## Introduction

Pediatric rheumatic diseases (PRDs) are a group of chronic inflammatory conditions characterized by periods of disease flare-ups and often accompanied by pain [1]. Pain in PRD is a common problem, with a prevalence up to 86% in children with juvenile idiopathic arthritis (JIA) [2, 3]. Adolescents with pain reported experiencing reduced levels of physical functioning compared to patients with either mild or no pain, and they reported a significantly higher school absenteeism over the previous 6 months [4]. Acute musculoskeletal pain in PRD can often be attributed primarily to local inflammation; therefore, an anti-inflammatory treatment regime is a key therapeutic feature [5]. However, acute pain can progress into chronic musculoskeletal pain (CMP), even if the disease activity score is low [6, 7].

Once musculoskeletal pain becomes chronic, it often persists into adulthood [1, 8–10]. Children with CMP experience high levels of stress and are prone to anxiety and depression, which can in turn lead to increased pain and disability [11–15]. In addition, children with CMP often report sleep difficulties, including a lack of refreshing sleep and increased fatigue, further disrupting their social and academic development [4, 16–20]. Moreover, the impact of CMP is not confined to the individual patient, but can extend to the entire family and can have significant societal costs [21–24].

Pain can negatively influence our behavior, activity, and participation; although this might be initially helpful, it can lose purpose if the pain becomes chronic. Acute pain is induced by local inflammation or injury. After that, peripheral and central sensitization contributes to an amplification of a new pain stimulus [25]. Finally, endogenous pain modulatory pathways determine pain responses by the influence of attention, suggestion, expectation, stress, anxiety, context and past experience [25]. While most pharmacological interventions are targeted on treatment of inflammation, alleviation of chronic pain might need another approach [3, 6, 7, 16]. Several studies found that in addition to biological processes, psychosocial factors such as coping and cognitive health beliefs can determine the experience and impact of chronic pain, giving rise to the so-called biopsychosocial model [15, 21, 26]. Expanding our knowledge beyond pharmacological solutions to include non-pharmacological interventions, such as psychological or exercise-based interventions, may, therefore, provide a promising means to alleviate pain and improve functioning in children with PRD.

Psychological therapies and exercise-based therapies have been shown to be beneficial for children with widespread chronic pain, who did not have PRD [27–30]. These therapies have also been shown to exert modest beneficial effects in adults with rheumatic disease [31]. In their review published in 2013, Cunningham and Kashikar-Zuck proposed that a multidisciplinary approach consisting of carefully selected pharmacological and non-pharmacological interventions based upon a biopsychosocial framework may provide the most effective approach to treating pain [31].

Although non-pharmacological therapies may represent a promising addition and/or alternative to pharmacological therapies for alleviating chronic pain, evidence with respect to using non-pharmacological therapies for CMP in children and adolescents with PRD is extremely limited. In this review, the aim is to provide an overview of published non-pharmacological therapies for CMP in patients with PRD. This overview may serve as a stepping stone for future research and for the implementation of non-pharmacological therapies in clinical practice.

## Methods

In our review, both randomized controlled trials (RCTs) and non-randomized controlled trials were eligible. The primary outcome measure was pain intensity, and the secondary outcome measures were functional disability and quality of life. We performed a systematic search of PubMed (both MEDLINE-indexed and PMC-archived items), Embase (Scopus), PsycINFO, and the Cochrane Library, with no date or language restrictions. The search terms included terms related to musculoskeletal pain or dysfunction, non-pharmacological treatment modalities, children, and pediatric rheumatic diseases. In addition, the reference lists of the retrieved papers were manually cross-referenced, and Scopus was used to search for additional relevant studies. The following inclusion criteria were used: (1) children 5–18 years of age with PRD and chronic musculoskeletal pain (defined as ≥ 3 months in duration) not associated with active disease; (2) it concerned primary research and was available in full-text; (3) the study included at least one non-pharmacological intervention arm such as exercise, physiotherapy, cognitive behavioral therapy (CBT), occupational therapy, biofeedback, or complementary and alternative medicine; and (4) the study outcomes included pain intensity. Exclusion criteria were: (1) Treatment arm with < 5 patients at the end of treatment; (2) studies on complex regional pain syndrome (CRPS); and (3) CMP associated with a malignant disease process.

The methodological quality of each included study was independently assessed by two authors (LNN and MMN) based on the Cochrane risk of bias tool, and the quality of evidence was assessed using the GRADE (Grading of Recommendations Assessment, Development and Evaluation) approach [32]. GRADE was developed to assess pooled data from studies in comparable settings, with comparable outcome parameters. In our review, however, the studies were separately assessed due to the limited numbers of studies available and the heterogeneity among these studies. Quality of evidence was categorized as very low, low, moderate, or high [32].

## Results

### Search results

**Fig. 1:**
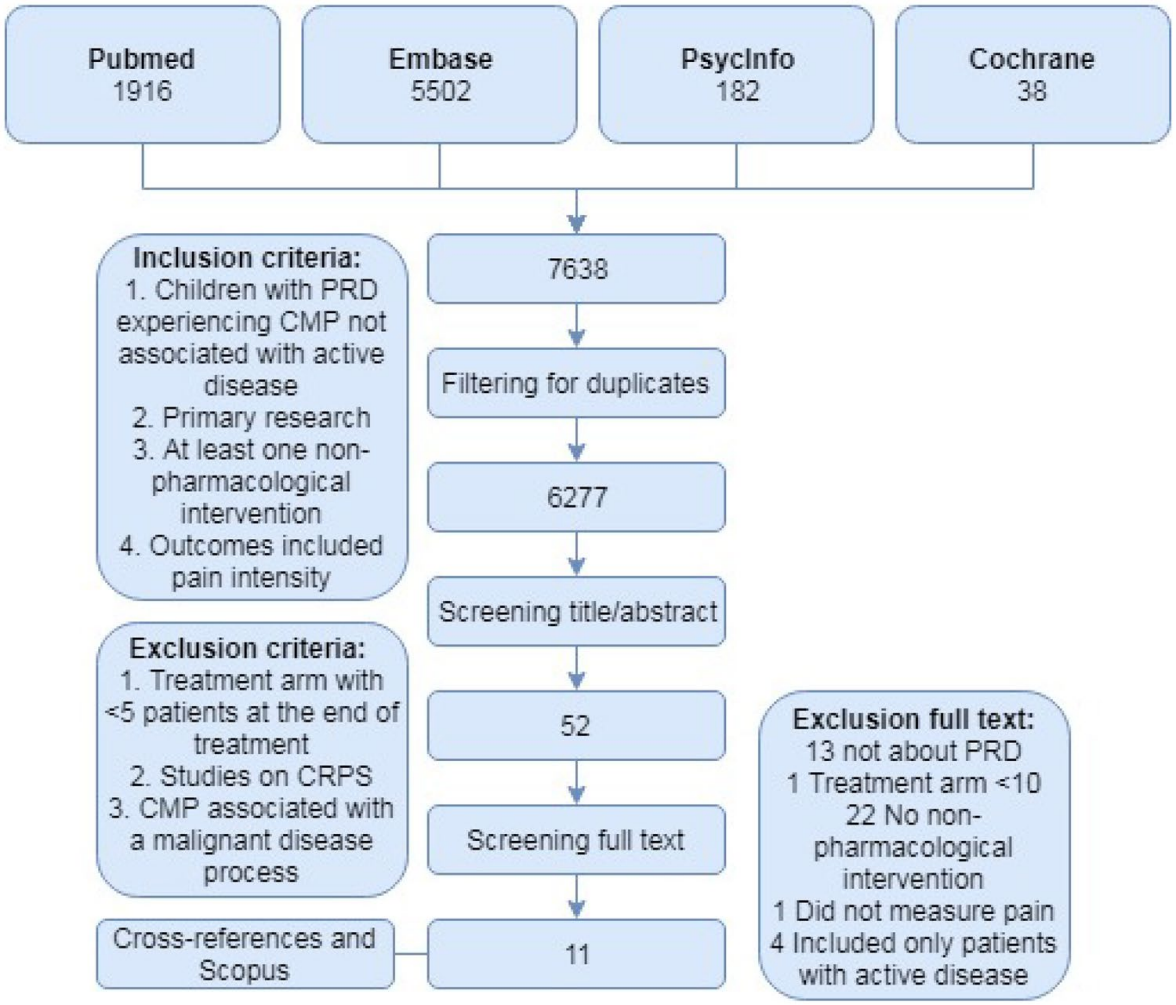
Flow-chart depicting the search strategy, inclusion and exclusion criteria, and studies included in the final analysis

The databases PubMed, Embase, PsycINFO, and the Cochrane Library were systematically searched for articles published through October 25, 2017, yielding a total of 7638 publications. After adjusting for duplicates, 6277 publications remained, of which 6225 were excluded after reviewing the title and/or abstract. After screening the full-text articles for the remaining 52 publications, we identified eleven randomized/non-randomized controlled trials that described non-pharmacological therapies for treating chronic pain in PRD (Fig. 1) [33–43]. Ten of these studies involved children with juvenile idiopathic arthritis (JIA), and one study involved children with systemic lupus erythematosus (SLE). The characteristics of the included studies are summarized in Supplementary Table S1.

Next, we attempted to differentiate between patients who had chronic pain with active disease and patients who had chronic pain in the absence of active disease. In our differentiation, we excluded the studies published by Epps et al. (2008), Singh-Grewal et al. (2007), Sandstedt et al. (2013), and Ramelet et al. (2017), as they included patients who were recently diagnosed or had active inflammation at the time of the intervention [44–47]. However, the study by Baydogan et al. included patients with active disease; in contrast, the description of active disease was not always conclusive for the remaining studies [35].

### Quality of the evidence

The eleven studies included in our review involved a total of 420 participants. The mean percentage of female patients in the studies was 71% (range 54–100%). All eleven studies had a relatively small cohort and were short-term studies; only two studies included more than 50 participants, and only one study reported follow-up data. Table 1 summarizes the GRADE evidence profiles. Overall, the quality of evidence ranged from very low to moderate.

**Table 1:** GRADE evidence profiles per study

No of participants included in the analysis	Summary of findings for pain			*P* value (posttreatment between groups)				Quality assessment																	
	Mean difference pre-post treatment (VAS score 0–10)		*P* value (pre-posttreatment per intervention)					Risk of bias						Indirectness					Imprecision						Overall quality of evidence
Physical therapy																									
Field et al. [42] massage therapy vs. relaxation therapy																									
Massage *N* = 10		− 3.0	< 0.005	Not given					Very serious^a^					Serious^f,g^					Serious^j^						⨁◯◯◯ Very low
Relaxation *N* = 10		− 0.5	NS																						
Klepper [38] physical conditioning program vs. waiting list																									
Physical conditioning *N* = 25		− 0.5	NS	Not applicable (within subjects design)					Very serious^b^					Not serious					Serious^k^						⨁◯◯◯ Very low
Waiting list *N* = 25		− 0.7	NS																						
Tarakci et al. [39] land-based home exercise vs. waiting list																									
Land-based home exercise *N* = 43		− 0.9	< 0.001	0.29					Not serious					Not serious					Serious^k^						⨁⨁⨁◯ Moderate
Waiting list *N* = 38		− 0.7	0.002																						
Mendonca et al. [41] pilates exercise vs. conventional exercise program																									
Pilates exercise *N* = 25		− 2.3	< 0.01	< 0.0001					Not serious					Not serious					Serious^k^						⨁⨁⨁◯ Moderate
Conventional exercise program *N* = 25		+ 0.2	NS																						
Baydogan et al. [35] strengthening vs. balance-proprioceptive exercise																									
Strengthening exercise *N* = 15		− 1	< 0.001	0.502					Serious^c^					Serious^g^					Serious^l^						⨁◯◯◯ Very low
Balance-proprioceptive exercise *N* = 15		− 1	< 0.001																						
Elnaggar and Elshafey [37] resistive underwater exercise vs. traditional physical therapy																									
Resistive underwater exercise *N* = 15		− 4.4	0.001		0.001				Not serious					Serious^h^					Not serious						⨁⨁⨁◯ Moderate
Traditional physical therapy *N* = 15		− 1.1	0.001																						
Psychological interventions																									
Stinson et al. [40] managing arthritis online program vs. attention control																									
Managing arthritis online *N* = 22		− 0.6	Not given		0.03				Not serious					Serious^f,g^					Not serious						⨁⨁⨁◯ Moderate
Attention control *N* = 24		+ 0.5	Not given																						
Brown et al. [43] cognitive behavioral therapy vs. education only vs. no contact																									
Cognitive behavioral therapy *N* = 22		Not given	Not given			CBT and education only vs. no-contact P = 0.68			Serious^d^					Serious^f,g^					Serious^l^						⨁◯◯◯ Very low
Education only *N* = 10		Not given	Not given																						
No-contact control *N* = 16		Not given	Not given																						
Lomholt et al. [34] cognitive behavioral therapy vs. waiting list																									
Cognitive behavioral therapy *N* = 9		+ 0.4	Not given			0.81			Not serious					Serious^i,g^					Serious^j^						⨁⨁◯◯ Low
Waiting list *N* = 10		+ 0.5	Not given																						
Eid et al. [36] physical therapy with biofeedback vs. physical therapy																									
Physical therapy with biofeedback *N* = 18		− 3.7	0.0001			0.001			Not serious					Serious^i,g^					Not serious						⨁⨁⨁◯ Moderate
Conventional physical therapy *N* = 18		− 2.2	0.0001																						
Spiegel et al. [33] peer support vs. waiting list																									
Peer support *N* = 16		− 0.3	Not given			0.63			Serious^e^					Serious^f,g^					Serious^k^						⨁⨁◯◯ Low
Waiting list *N* = 14		− 0.2	Not given																						

*NS* not significant

^a^Random sequence generation, allocation concealment, attrition bias and reporting bias not described

^b^There was risk of selection, attrition and detection bias

^c^There was risk of detection bias, allocation concealment was not described

^d^There was risk of attrition bias, allocation concealment was not described

^e^There was risk of attrition bias, blinding of outcome assessment was not described

^f^Only adolescents

^g^The vast majority was female

^h^No information was given concerning age range and gender of participants

^i^Limited age range

^j^Small sample size

^k^Very large standard deviation. l. no information was given regarding standard deviation or confidence interval

### Effectiveness of psychological interventions

Several studies reported a moderate reduction in pain with the addition of biofeedback to physical therapy and an online program online for self-management and education; these studies assessed pain using a visual analog scale (VAS) [36, 40]. In contrast, participating in a peer-support program did not result in a significant decrease in pain (measured using the recalled pain inventory) compared to control patients [33]. Two studies measured the effect of cognitive behavioral therapy (CBT), in patients with JIA and patients with SLE and found no difference in either pain or quality of life compared to the respective control groups; both of these studies assessed pain using a VAS, and one additionally used the McGill Pain Questionnaire [34, 43]. With respect to studies that reported functional disability (assessed using Child Health Assessment Questionnaire, the Functional Disability Inventory, or the Juvenile Arthritis Functional Assessment Report), functioning was improved with biofeedback, but not with CBT or telephone consultation with a nurse [36, 40]. Quality of life (measured using the PedsQL or the Juvenile Arthritis Quality of life Questionnaire) did not differ between patients who received peer support, or the online arthritis managing program compared to patients in the respective control groups [33, 40].

### Effectiveness of physical therapy

A significant decrease in pain was reported following massage therapy, but not following relaxation therapy [42]. In addition, Tarakci et al. reported that although a 12-week exercise-based intervention significantly reduced pain (the mean change in VAS was − 0.9), the control group had a similar reduction in pain (with a mean change in VAS of − 0.7); however, exercise was more effective at improving both functional capacity and quality of life [39]. They used a combination of strengthening, stretching and postural exercises and functional activities (walking, squat and stair-climbing). In contrast, Klepper reported that pain levels did not change following an exercise intervention, using low-impact aerobic exercise to improve aerobic endurance, muscular strength, and flexibility [38]. Both strength-building exercises (focused on the quadriceps femoris and hamstrings) and balance-proprioceptive exercises were equally effective at reducing pain and functional disability [35]. Another study found that Pilates exercise, but not conventional exercise (described as mainly stretching exercises and improving core stability), significantly reduced pain, improved function, and increased quality of life [41]. Combined resistive underwater exercise and traditional physical therapy were both found to reduce pain, but the underwater exercises were more effective [37]. Traditional physical therapy consisted of hot packs, range-of motion, isometric and stretching exercises, and fitness exercises such as cycling and treadmill walking. All of the above-mentioned studies used a VAS pain scale, the CHAQ, and/or the PedsQL questionnaire to measure pain, functional disability, and quality of life, respectively.

### Effects at follow-up

Only one study, which involved adolescent girls with SLE, included follow-up data [43]. In their study, the authors found that patients in the CBT group did not differ significantly from patients in the education and no-contact control groups at either the 3-month or 6-month follow-up time points.

## Discussion

### Main findings

Chronic musculoskeletal pain is relatively common in patients with pediatric rheumatic disease and can be highly debilitating. Despite the high impact that CMP can have on the patient’s functioning and social participation, therapeutic options are limited. The strikingly few studies involving non-pharmacological therapies reported modest beneficial results in response to psychological and exercise-based interventions. On the other hand, some studies found no clear benefits associated with active non-pharmacological treatments with respect to reducing pain or improving function. This discrepancy may have been due—at least in part—to the difficulty differentiating between acute and chronic pain among the patients in the included studies. Importantly, none of the studies reported side effects associated with the non-pharmacological therapies, making this approach a potentially promising alternative or addition when pharmacological therapies are insufficient for alleviating pain.

### Comparison with previous reports

Various aspects of non-pharmacological therapies for CMP in patients with PRD have been discussed previously. For example, Cohen et al. (2017) recently performed a meta-analysis to review the effect of psychosocial therapies on pain in PRD [48]. However, their search was focused on psychosocial interventions and included five articles, two of which were studies involving fibromyalgia. The authors concluded that the results were, therefore, too limited to draw any meaningful conclusions. Similarly, both Takken et al. (2008) and Kuntz et al. (2018) published a review regarding the effect of exercise therapy in JIA [49, 50]. Although both groups reported that exercise therapy appears to be well tolerated and beneficial in terms of reducing pain and improving the function and quality of life among patients with JIA, they noted that specific clinical recommendations may be premature. One possible explanation for their inconclusive results is the heterogeneity among the patients included in the studies.

### Strengths and limitations

In all three above-mentioned reviews, no distinction was made between acute and chronic pain or between the presence or absence of active inflammation; importantly, this differentiation might provide insight into which approach might be most effective. In this respect, a strength of our review is our attempt to distinguish among these different patient groups, as these different groups may require different approaches. One limitation is that non-pharmacological therapies include a broader range of interventions than just psychological and exercise-based interventions. Many areas of non-pharmacological treatment modalities such as chiropractic treatment and mindfulness have not been evaluated in a controlled trial or focus on non-specific generalized chronic pain and, therefore, were not included in our systematic review [51, 52]. Second, the treatment effect of some interventions could not be addressed fully, as in some cases they were compared to another intervention (for example, one form of exercise vs. another form of exercise). Third, the currently available evidence was too limited to pool the data or perform a meta-analysis. Finally, we were unable to adjust our result for the sex or age of the patients in the included studies. In general, pain tends to be more prevalent among girls, and this increases with age [53]. It is possible that there are age or gender specific differences in the responses to non-pharmacological therapies that we are not aware of.

### Implications for future research

With regard to future research, several aspects are worth mentioning. First, future studies should differentiate between acute and chronic pain, as these two types of pain differ with respect to the underlying pathophysiology. Second, studies that combine a graded exercise therapy with cognitive behavioral interventions to achieve change in the perception of pain might be a feasible approach for restoring functional capacity, increasing social participation, and reducing pain [31]. Third, based on the biopsychosocial model, we hypothesized that CBT may be highly effective; however, we were unable to test this hypothesis due to the limited number of studies available. Thus, gaining further insight into the relationship between an individual child’s thinking, feeling, and behavior might be necessary to tailor the cognitive behavioral intervention to that particular child for the psychological intervention to be effective. Finally, even though follow-up studies are extremely important, only one of the eleven studies in our analysis reported follow-up data (in this case, 6 months of follow-up). An improvement in functioning often precedes a reduction in pain [54]. Therefore, any reduction in pain might be more evident at later time points than immediately following treatment. Given that most of the studies in our analysis were published in 2010 or more recently, follow-up data may still be on the way, and opportunities for future research are numerous in this still-evolving field.

## Conclusions

Both psychological and exercise-based interventions have been shown to have modest beneficial effects on CMP in PRD. Moreover, non-pharmacological therapies are not associated with side effects. When pharmacological therapy is insufficient to alleviate pain in PRD, non-pharmacological therapies may serve as a suitable alternative and/or addition for reducing CMP and improving function. Importantly, chronic pain and acute pain may be etiologically different in PRD, and future studies should take this difference into account to identify the optimal therapeutic window for non-pharmacological approaches. Finally, studies are needed that specifically investigate chronic pain in PRD and are designed to improve social participation in children with PRD-related chronic pain, particularly with respect to the long-term effectiveness of these interventions.

## Electronic supplementary material

Below is the link to the electronic supplementary material.

Supplementary material 1 (DOC 46 KB)

## Abbreviations

CBT: Cognitive behavioral therapy
CHAQ: Child Health Assessment Questionnaire
CI: Confidence interval
CMP: Chronic musculoskeletal pain
FDI: Functional disability inventory
JAFAR-C: Juvenile Arthritis Functional Assessment Report
JAQQ: Juvenile Arthritis Quality of Life Questionnaire
JIA: Juvenile idiopathic arthritis
JRA: Juvenile rheumatoid arthritis
PedsQL: Pediatric quality of life inventory
PRD: Pediatric rheumatic disease
RCT: Randomized controlled trial
SLE: Systemic lupus erythematous
VAS: Visual analog scale
