# Supplementary material for: Non-pharmacological options for managing chronic musculoskeletal pain in children with pediatric rheumatic disease: a systematic review
Source: Rheumatol Int. 2018 Aug 23;38(11):2015–25. doi: 10.1007/s00296-018-4136-8 (PMC6208689; doi:10.1007/s00296-018-4136-8)
Supplement: Supplementary file 1 — Supplementary material 1 (DOC 46 KB) [file 296_2018_4136_MOESM1_ESM.doc]

**Supplementary Table S1.** *Summary of the characteristics of the eleven studies included in the review*

| **Study** | **Primary diagnosis** | **Participants** | **Intervention** | **Duration of treatment** | **Outcome measure pain** | **Outcome measure functional disability** | **Outcome measure quality of life** |
| --- | --- | --- | --- | --- | --- | --- | --- |
| Field et al. (1997) | JRA | 5-15 years  70% female | Massage therapy vs. relaxation therapy | Frequency: 15 min/day at bedtime  Duration: 30 days | VAS pain  PPQ-Child Form  PPQ-Parent form  Number of severe pain points  Words for pain | - | - |
| Klepper (1999) | JRA | 8-17 years  92% female | Physical conditioning program vs. waiting list | Frequency: 60 min sessions 3x/week, once at home  Duration: 8 weeks | VAS pain | - | - |
| Stinson et al. (2010) | JIA | 12-18 years  70% female | Managing arthritis online program vs. attention control | Frequency: Intervention group one module/week (30 min) and coaching via phone calls vs. coaching via phone calls concerning self-management  Duration: 12 weeks | VAS pain  Recalled Pain Inventory | - | JAQQ |
| Brown et al. (2012) | SLE | 12-18 years  100% female | CBT vs. education only/no-contact | Frequency: 3 sessions, 3x 45 min lessons at home and 2 telephone calls  Duration: 6 weeks | VAS pain  McGill Pain Questionnaire – Short Form | - | PedsQL |
| Tarakci et al. (2013) | JIA | 5-17 years  54% female | Land-based home exercise vs. waiting list | Frequency: 1 day/week under physical therapist’s supervision, 3 days/week under parent’s supervision for 20-45 minutes  Duration: 12 weeks | VAS pain | CHAQ | PedsQL |
| Mendonça et al. (2013) | JIA | 8-18 years  64% female | Pilates exercise vs. conventional exercise program | Frequency: 50 min session 2x/week  Duration: 24 weeks | VAS pain | CHAQ | PedsQL |
| Baydogan et al. (2015) | JIA | 6-18 years  70% female | Strengthening vs. balance-proprioceptive exercise | Frequency: 45 min sessions, 3x/week supervised, 4x/week at home  Duration: 12 weeks | VAS pain | CHAQ | - |
| Lomholt et al. (2015) | JIA | 9-14 years  79% female | CBT vs. waiting list | Frequency: 120 min sessions, 1x/week  Duration: 6 weeks | VAS pain | FDI | PedsQL |
| Eid et al. (2016) | JRA | 8-13 years  69% female | Physical therapy with biofeedback (n=18) vs physical therapy (n=18) | Frequency: 60 min/session 3x/week (in the study group 15 min/session biofeedback)  Duration: 12 weeks | VAS pain | JAFAR-C[53] | - |
| Elnaggar et al. (2016) | JIA | Age range not provided  % female not given | Resistive underwater exercise vs. traditional physical therapy | Frequency: 45 min session 3x/week  Duration: 12 weeks | VAS pain | - | - |
| Spiegel et al. (2017) | JIA | Children aged 12-18 years old  94% female | Peer support and education vs. control | Frequency: 10 sessions of 20-30min video calls  Duration: 8 weeks | Recalled Pain Inventory | - | PedsQL Arthritis Module |
| ACR: CBT: cognitive behavioral therapy; CHAQ: Child Health Assessment Questionnaire; FDI: Functional Disability Inventory; JAFAR-C: Juvenile Arthritis Functional Assessment Report; JAQQ: Juvenile Arthritis Quality of Life Questionnaire; JIA: juvenile idiopathic arthritis; JRA: juvenile rheumatoid arthritis; PedsQL: Pediatric Quality of Life Inventory; SLE: systemic lupus erythematous; VAS: visual analog scale. | | | | | | | |
